# Decreased Frequency of Splenectomy for High-Grade Splenic Injuries Following the Implementation of an Interventional Radiology Alert Protocol

**DOI:** 10.7759/cureus.114060

**Published:** 2026-08-06

**Authors:** Maura Kopchak, John Bach, William B DeVoe, Brian Dusseau, Katherine A Graham, John Dillis, Thomas Glusich, Jeff Hubartt

**Affiliations:** 1 Surgery, OhioHealth Riverside Methodist Hospital, Columbus, USA; 2 General Surgery, OhioHealth Riverside Methodist Hospital, Columbus, USA; 3 Surgery, OhioHealth Research Institute, Columbus, USA; 4 Trauma, OhioHealth Riverside Methodist Hospital, Columbus, USA

**Keywords:** hemorrhage, interventional radiology guided embolization, splenectomy, splenic embolization, splenic trauma

## Abstract

Background

A vascular interventional radiology (VIR) protocol was initiated at a mature level 2 trauma center on 3/1/2023 to reduce time to vessel puncture to < 60 minutes as per American College of Surgeons (ACS) guidelines. The average time to vessel puncture in the two years prior to protocol initiation was 102 minutes. Following initiation, time to vessel puncture was reduced to 48.2 minutes (p < 0.001). A statistically significant increase in the number of splenic embolizations was noted in the post-protocol group. The primary outcome of the current study was to evaluate if the initiation of a VIR protocol correlated with a statistically significant decrease in splenectomies for high-grade splenic injuries (American Association for the Surgery of Trauma (AAST) grade 3 or higher) in favor of splenic embolization. The secondary outcomes of the study were to assess whether the initiation of the VIR alert protocol improved our rate of splenic salvage, defined as preserved splenic immune function, and if there was a decreased rate of splenectomies across all injury grades.

Methods

The trauma registry database was queried for all splenic injuries from 1/1/2021 to 9/1/2024. Admission CT scans were used to grade injuries using the AAST Organ Injury Scale. Demographic and clinical characteristics, including severity of illness (ISS), admission INR, age, blood product required pre- and post-intervention, mortality, sex, length of stay, anticoagulant (AC)/antiplatelet (AP) use, mechanism of injury, and need for REBOA (resuscitative endovascular balloon occlusion of the aorta) placement, were collected for all patients. Patients were further broken down into those requiring splenectomy, embolization, or no intervention. Data were analyzed using chi-square and Mann-Whitney U with Jamovi Version 2.3.26.0 (The Jamovi Project, 2023).

Results

No statistically significant difference was found for clinical or demographic characteristics between groups. Fewer splenectomies and more splenic embolizations were performed in the post-protocol group for high-grade splenic injuries (grade 3), which was statistically significant (p<0.001). This was also true when all grade splenic injuries were compared (p<0.001).

Conclusion

Following the establishment of an institutional VIR alert protocol for active hemorrhage control in trauma patients, more frequent utilization of angioembolization for high-grade splenic injuries was found with a subsequent decrease in splenectomy rate. Implementation of the VIR alert protocol directly correlated with a shift in practice pattern reflecting attempts at splenic salvage for all injury grades.

## Introduction

Traumatic injuries are a leading cause of death in the United States, and uncontrolled hemorrhage is responsible for 30-40% of all trauma mortality [1,2]. Solid organ injury is a frequent cause of uncontrolled hemorrhage, with splenic injury being the most common, with over 40,000 adults suffering spleen injuries each year [3]. With the development of improved computerized tomography (CT) and endovascular embolization techniques, management of active hemorrhage has evolved to include multiple disciplines, including interventional radiology (IR) [4]. Angioembolization is a diagnostic and therapeutic intervention used to control bleeding from splenic injuries and is an important adjunct for improving splenic salvage rate [5].

The American College of Surgeons Committee on Trauma (ACS-COT) recommends that all verified Levels I and II trauma centers achieve vessel puncture within 60 minutes of the decision for angiography for active bleeding best managed via interventional modalities [6,7]. To help meet this requirement at a high-volume Level II trauma center, an institutional protocol was developed to improve communication across a multidisciplinary team consisting of trauma surgery, interventional radiology, and emergency medicine when an appropriate patient is deemed best managed with angioembolization for active hemorrhage. The protocol starts when an appropriate hemorrhage is identified on CT imaging. The Trauma Attending then calls the Interventional Radiology Attending to discuss the case. A mutual agreement is then made to call a vascular interventional radiology (VIR) alert. An alert is then paged out to the Emergency Department, Trauma team, and the Interventional Radiology staff. The use of this protocol allowed for multiple processes to occur simultaneously and with more efficiency, demonstrating decreased time to vessel puncture and, ultimately, hemorrhage control by an average time of 48 minutes in the pilot study [8].

Through continuous process improvement efforts, the effect of the alert protocol has continued to be evaluated primarily for time to vessel puncture and for additional clinical outcomes. A lower frequency of splenectomies was observed in the post-protocol implementation group, indicating an increased splenic salvage rate. The primary outcome of the current study was to evaluate if the initiation of a VIR protocol correlated with a statistically significant decrease in splenectomies for high-grade splenic injuries (AAST grade 3 or higher) in favor of splenic embolization. The secondary outcomes were to assess whether the initiation of the VIR alert protocol improved our rate of splenic salvage, defined as preserved splenic immune function, and to observe if there was a decreased rate of splenectomies across all injury grades.

## Materials and methods

**Figure 1:**
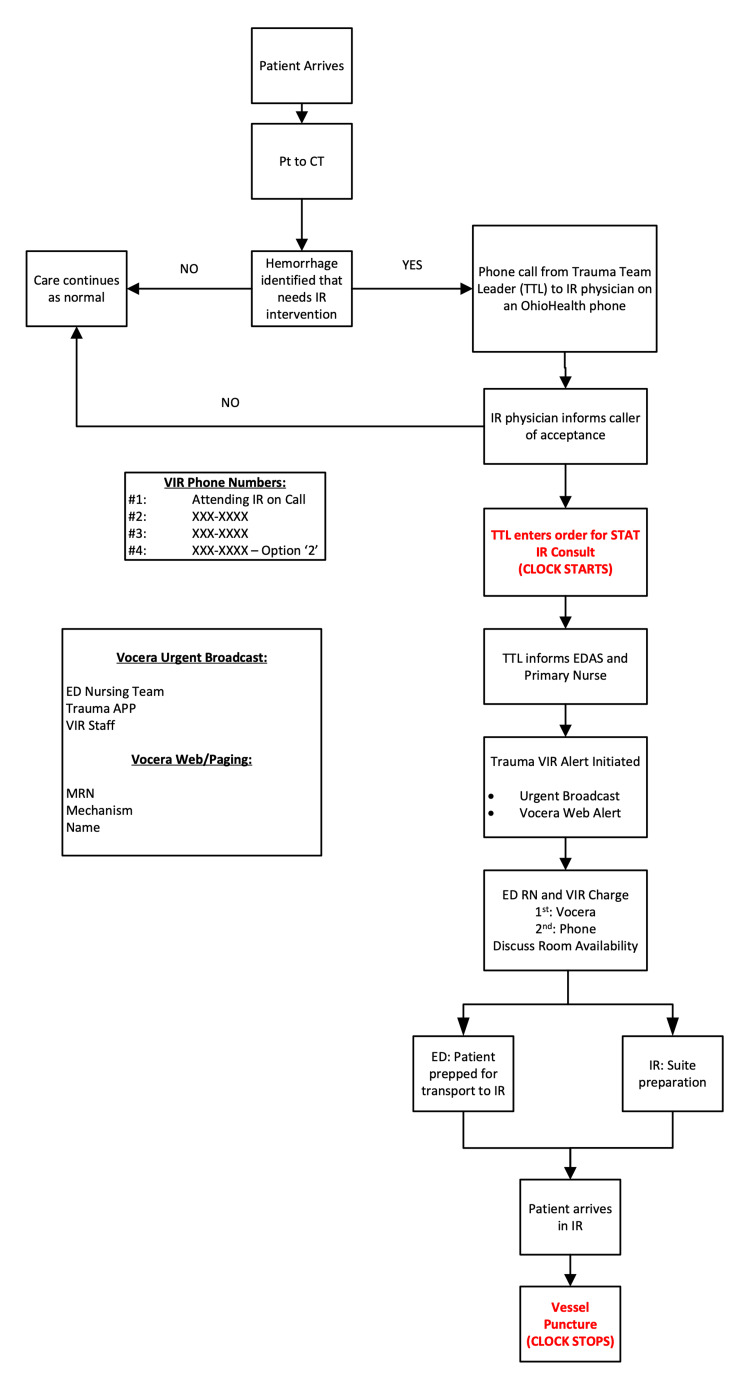
IR alert protocol IR: interventional radiology

**Figure 2:**
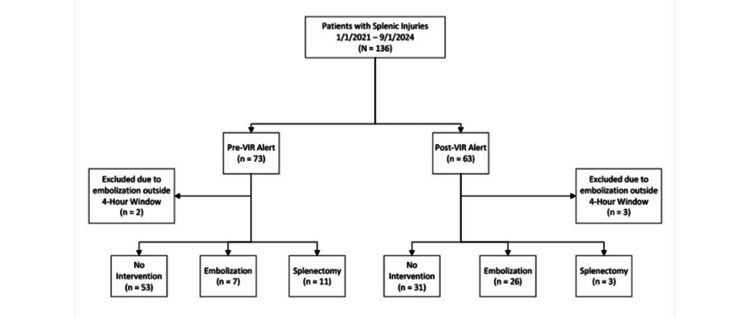
Inclusion flowchart

A retrospective review of our institution’s trauma registry was performed on all trauma patients admitted to a Level II center with splenic injuries between 1/1/2021 and 9/1/2024. The trauma registry consists of all trauma patients at our center and contributes to the American College of Surgeons Trauma Quality Improvement Program (ACS-TQIP) registry [9]. The IR alert protocol (Figure 1) was formally initiated in March of 2023. Patients were divided into pre- and post-IR alert cohorts, and then classified into those receiving either no intervention, angioembolization, or trauma splenectomy (Figure 2). Patients who received angioembolization beyond four hours of presentation were excluded. The four-hour threshold was chosen to represent all patients deemed to require a procedure either emergently or urgently based on their injury and physiology at presentation. Using this criterion, two and three patients were excluded from the pre- and post-alert groups, respectively.

Splenic injuries were graded using admission CT images by on-call attending radiologists according to the American Association for the Surgery of Trauma (AAST) Organ Injury Scale (OIS) spleen injury scale [6]. The associated charts were reviewed, and data were collected for patient age, sex, mechanism of injury, severity of illness (ISS), length of stay, anticoagulation and antiplatelet status, admission INR, blood product utilization before and after intervention, need for REBOA (resuscitative endovascular balloon occlusion of the aorta) placement, and mortality. The total number of units of whole blood and packed red blood cells was used to define blood utilization. Notably, all patients included had results for all studied variables. Any patients with missing variables were excluded from analysis.

Demographic data were analyzed, predominantly using the chi-square analysis; however, age and ISS were analyzed using the t-test and Mann-Whitney U test, respectively. The distribution of each injury grade undergoing no intervention, embolization, and splenectomy pre- and post-IR protocol was compiled. A chi-squared analysis was performed, comparing the pre-IR alert rate of splenectomy and embolization with the equivalent groups in the post-IR alert grouping for all grades of splenic injuries. Then, to further assess this relationship in higher-grade injuries who would be at elevated risk of needing intervention, a second chi-squared analysis was performed including only high-grade (AAST of 3 or greater) injuries. All data were analyzed using Jamovi Version 2.3.26.0 (The Jamovi Project, 2023). We adhered to the standards for quality improvement reporting excellence (Squire 2.0) guidelines [10].

## Results

In total, 136 patients were identified in the study period. Of the 75 total splenic injuries observed in the 24 months preceding protocol initiation, 19 required interventions. In the 18 months following protocol implementation, 63 total splenic injuries were observed, with 29 requiring intervention. Patient demographic data for those undergoing intervention, either angioembolization or splenectomy, are shown in Table 1. Other than a higher proportion of female patients in the post-alert group, there were no additional statistically significant differences in age, ISS, or mechanism of injury between groups. The clinical characteristics between cohorts are described in Table 1. Initial INR, amount of blood product required, length of stay, mortality, and need for REBOA deployment were also not statistically significant between the pre- and post-IR-alert groups. Notably, there was no difference in the percentage of high-grade (≥ 3) splenic injuries in the pre- and post-IR alert groups.

**Table 1:** Demographics and clinical characteristics Mann-Whitney U test used for age and ISS. Chi-squared test used for the remainder. MVC: motor vehicle crash; GSW: gunshot wound; MCC: motorcycle crash; ISS: severity of illness; REBOA: resuscitative endovascular balloon occlusion of the aorta; LOS: length of stay

Characteristic	Pre-IR Alert (n=18)	Post-IR Alert (n=29)	p value	test
Age, median (range)	51 (24-76)	62 (20-85)	0.375	U = 220.00
Sex, n(%)	Pre-IR Alert	Post-IR Alert	p value	test
Female	3 (16.67)	13 (44.83)	0.048	X² = 3.92
Male	15 (83.33)	16 (55.17)		
ISS, median (range)	25 (4-54)	26 (5-43)	0.913	U = 255.50
Mechanism, n(%)	Pre-IR Alert	Post-IR Alert	p value	test
Assault	0 (0.00)	2 (6.90)	0.121	X² = 11.42
Bike Accident	1 (5.56)	0 (0.00)		
Fall	4 (22.22)	15 (51.72)		
GSW	1 (5.56)	0 (0.00)		
MCC	1 (5.56)	3 (10.34)		
MVC	9 (50.00)	9 (31.03)		
Stabbing	1 (5.56)	0 (0.00)		
Misc	1 (5.56)	0 (0.00)		
Initial INR, median (range)	1.10 (1.00-2.30)	1.10 (0.90-2.50)	0.649	U = 240.50
Pre-Procedure Product, median (range)	1 (0-9)	1 (0-8)	0.596	U = 237.00
Post-Procedure Product, median (range)	0 (0-24)	0 (0-2)	0.117	U = 219.00
REBOA, n(%)	1 (5.56)	1 (3.45)	0.728	X² = 0.12
Mortality, n(%)	3 (15.79)	4 (13.79)	0.788	X² = 0.07
LOS (days), median (range)	6 (1-42)	6 (1-26)	0.456	U = 226.50
Grade Injury ≥3, n(%)	16 (88.89)	26 (89.66)	0.934	X² = 0.01

The distribution of patients’ grade of splenic injury and respective management (no intervention, angioembolization, or splenectomy) both before and after protocol initiation are demonstrated in Table 2. Overall, the majority of the low-grade splenic injuries (AAST grade 1 or 2) were managed without intervention for both groups. There were infrequent occurrences of embolization (n=1) or splenectomy (n=1) in the pre-alert cohort and a similar infrequent need for embolization (n=3), but not splenectomy, in the post-alert group for low-grade injuries. After additional chart review, the low-grade injuries embolized were in patients suspected to have active bleeding based on hemodynamics, inappropriate physiologic response to blood transfusion, or active contrast extravasation on CT angiography. The isolated splenectomy for a grade 2 injury was performed for active bleeding encountered intraoperatively following a stab wound to the left upper quadrant in combination with diaphragmatic repair. For high-grade splenic injuries, AAST 3 or greater, a shift in the number of patients undergoing angioembolization compared to splenectomy was appreciated in the period following the IR-alert process (pre-IR alert: angioembolization n=6, splenectomy n=10; post-IR alert: angioembolization n=23, splenectomy n=3). There was an absolute reduction in the need for splenectomy in the post-IR alert group, with splenectomy only being performed for grade 5 injuries during the study interval. There were no occurrences of delayed splenectomy after embolization during the index hospitalization.

**Table 2:** Splenic injury grade and associated intervention pre and post-IR alert IR: interventional radiology

Pre-IR Alert				Post-IR Alert		
Splenic Injury Grade, N (%)	No Intervention (n=53)	Embolization (n=7)	Splenectomy (n=11)	No Intervention (n=31)	Embolization (n=29)	Splenectomy (n=3)
1	19 (35.85)	0 (0.00)	1 (9.10)	14 (45.16)	1 (3.85)	0 (0.00)
2	23 (44.23)	1 (14.29)	0 (0.00)	13 (41.94)	2 (7.69)	0 (0.00)
3	10 (19.23)	4 (57.14)	4 (36.36)	2 (6.45)	7 (26.92)	0 (0.00)
4	1 (1.92)	2 (28.57)	4 (36.36)	2 (6.45)	7 (26.92)	0 (0.00)
5	0 (0.00)	0 (0.00)	2 (18.18)	0 (0.00)	9 (34.62)	3 (100.00)

A chi-squared analysis was performed to assess the effect of the IR-alert process on splenic salvage rates. For all injury grades, there was a statistically significant (p < 0.001) increase in embolization utilization and decrease in splenectomy frequency pre- versus post-IR alert (Table 3). For AAST 3 or greater splenic injuries, this remained statistically significant (p<0.001), again demonstrating more frequent use of angioembolization over splenectomy for higher-risk injuries (Table 4).

**Table 3:** Intervention: all splenic injury grades Chi-squared test used

Intervention	Pre-alert (n=18)	Post-alert (n=29)	p value	test
Embolization, n(%)	7 (38.89)	26 (89.66)	<0.001	X² = 13.69
Splenectomy, n(%)	11 (61.11)	3 (10.34)	-	-

**Table 4:** Intervention: high-grade splenic injuries Includes grade 3-5 injuries; chi-squared test used

Intervention	Pre-alert (n=16)	Post-alert (n=26)	p value	test
Embolization (grade≥3), n(%)	6 (37.50)	23 (88.46)	<0.001	X² = 12.04
Splenectomy (grade≥3), n(%)	10 (62.50)	3 (11.54)	-	-

## Discussion

A multidisciplinary team-based approach is critical to the diagnosis and treatment of the actively bleeding patient [11]. It is well-documented that delays in intervention are generally due to lack of appropriate, timely communication between consulting and specialist teams [12-14]. These inefficiencies can exacerbate uncontrolled hemorrhage, leading to unintended outcomes, including worsening shock state, increased blood product requirement, and prolonged hospital stay [12,14]. Through multidisciplinary collaboration, a previously developed and enacted protocol to obtain endovascular hemorrhage control in actively bleeding trauma patients led to a significant reduction in time to vessel puncture from the decision for angiography from 102 to 48 minutes [7]. This decrease in time to vessel puncture is made possible by 24/7 vascular interventional radiology and trauma coverage at this institution. This decreased time to hemorrhage control was appreciated in patients with similar ISS, mechanism of injury, pre-procedure blood product transfusion, antiplatelet therapy, and anticoagulation in the pilot study. The process improved communication between the trauma service, emergency department, and interventional radiology by setting expectations among team members while streamlining communication regarding patients with active hemorrhage who are appropriate candidates for embolization. Further, the protocol allowed for multiple steps to occur in parallel after activation, ultimately resulting in more rapid time to endovascular intervention and hemorrhage control (Figure 1).

Endovascular treatment of blunt solid organ injuries has been associated with organ preservation, survival, and other improved clinical outcomes. Prompt endovascular intervention plays a crucial role in controlling hemorrhage in trauma patients when angioembolization is an appropriate treatment modality [4]. Studies have suggested that splenic artery embolization is a safe and effective management approach for hemodynamically stable high-grade splenic injuries. More recent studies have also demonstrated that hemodynamically unstable patients may be acceptably managed with embolization [15]. This evaluation found that a recently developed IR alert protocol led to a statistically significant increase in the percentage of splenic injuries undergoing splenic embolization versus splenectomy in the pre/post protocol analysis. There was no difference in ISS, blood product utilization, and mortality between groups, with a similar percentage of high-grade (AAST 3 or greater) injuries between cohorts. These results demonstrate the implementation of an IR protocol to have had a significant impact on splenic salvage for high-grade splenic injuries within a Level II trauma center.

Targeted embolization of solid organ injuries allows for organ preservation and improved organ function [16]. Benefits of splenic preservation include maintenance of splenic immune function and prevention of overwhelming infection from encapsulated organisms. Asplenia carries a risk of overwhelming post-splenectomy infection (OPSI) from encapsulated bacteria, which is associated with a 50% mortality and an overall lifetime risk of 0.1% to 8.5% [17-19]. Accordingly, angioembolization is advocated in appropriately selected injuries to avoid the risks of asplenia. Splenic artery embolization has been shown to be effective in treating hemodynamically stable patients who present with high-grade splenic injuries with decreased risk of early infectious complications [20]. Failure of angioembolization to control bleeding in blunt splenic injury occurs in 5% to 15% [20,21]. Our results support angioembolization in these high-grade splenic injuries without increased failure rates, negative outcomes, or occurrences of delayed splenectomy observed in the study period during the index hospitalizations.

There are several limitations to this study. The first is that these results suggest a correlational relationship between the implementation of the VIR alert protocol and a decreased rate of splenectomies but would not be sufficient to show a true causal relationship. The most significant limitation is the small sample size and retrospective nature of data collection for the pre-protocol implementation group. Although a decreased rate of splenectomy occurred in the post-protocol group, a decrease in blood utilization, length of stay, or mortality was not observed. The data set was also limited in that the extent of embolization (selective vs non-selective) in the study group was not well defined. There is also a potential for a “pre- and post-intervention” bias based on the study design. This was likely amplified through our trauma program educational interventions and multiple in-situ simulations of the IR alert protocol. Although no occurrences of embolization failure or related complications requiring delayed intervention were seen, it is important to acknowledge the bias for and potential of missed post-embolization failure or complications. This may be from nonoperative treatment selection bias, improved competency of interventional radiologists, patients presenting to other trauma centers following discharge, or unidentified admissions outside of the study period. It is also possible that due to the length of this study, we are underestimating the number of complications and failures associated with embolization. This is a future direction for further exploration as more data is gathered prospectively.

Areas for continued investigation are multiple. As previously described, the potential for overuse of embolization as a result of the protocol and post-embolization complications is possible and an area of future work. Further research is needed to determine whether the IR alert protocol results are sustainable and generalizable to other trauma centers. Additionally, whether the protocol effect extends to other clinical outcomes, such as blood product utilization or mortality, remains to be seen and will require further study with an increased sample size.

Following establishment of an institutional IR alert protocol for active hemorrhage control in trauma patients, more frequent utilization of angioembolization for high-grade splenic injuries was found with a subsequent decrease in splenectomy rate. Implementation of the IR alert protocol directly correlated with a shift in practice pattern, reflecting attempts at splenic salvage for all injury grades. Although no occurrences of delayed splenectomy were observed during the study period, this represents an important area for continued research given the limitations of the current study.

## Conclusions

Following the establishment of an institutional VIR alert protocol for active hemorrhage control in trauma patients, more frequent utilization of angioembolization for high-grade splenic injuries was found, with a subsequent decrease in the splenectomy rate. This relationship was also upheld across all injury grades. This relationship was independent of age, sex, ISS, mechanism of injury, initial INR, blood product utilization, length of stay, mortality, and REBOA utilization. Implementation of the VIR alert protocol directly correlated with a shift in practice pattern reflecting attempts at splenic salvage for all injury grades. Our results support angioembolization in high-grade splenic injuries without increased failure rates, negative outcomes, or occurrences of delayed splenectomy observed in the study period during the index hospitalizations.
